# Combination of immunotherapy and whole-brain radiotherapy on prognosis of patients with multiple brain metastases: A retrospective cohort study

**DOI:** 10.1515/biol-2025-1102

**Published:** 2025-09-01

**Authors:** Pengwei Yan, Changzhai Wang, Duixian Tuoligan, Aji Kabinuer, Sheng Li, Xue Song, Huanfeng Zhu

**Affiliations:** Department of Radiation Oncology, Jiangsu Cancer Hospital, the Affiliated Cancer Hospital of Nanjing Medical University, Jiangsu Institute of Cancer Research, No. 42 Baiziting, Xuanwu District, Nanjing, 210009, P.R. China; Department of Radiation Oncology, the Affiliated Kezhou People’s Hospital of Nanjing Medical University & People’s Hospital of Kizilesu Kirgiz Autonomous Prefecture, Kezhou, 845350, Xinjiang, P.R. China

**Keywords:** whole brain radiotherapy, integrated boost, immunotherapy, brain metastases

## Abstract

Brain metastases (BMs) usually occur in the advanced stage of cancers with a poor prognosis. This study aimed to compare the clinical efficacy and effects on cognitive function of immunotherapy combined with whole brain radiotherapy (WBRT) and immunotherapy combined with WBRT plus sequential integrated boost (SEB) in the treatment of multiple BMs. A total of 57 patients diagnosed with BMs were included in Kezhou People’s Hospital Affiliated to Nanjing Medical University between 2021 and 2023. Patients were allocated into the WBRT group (*n* = 27) and the WBRT + SEB group (*n* = 30) based on whether to receive a boost. The WBRT + SEB group showed a higher complete response rate and objective response rate compared to the WBRT group (26.7 vs 14.8%, 90.0 vs 66.7%) (all *P* < 0.05). The two groups had a median overall survival (OS) time of 11.2 months (95% confidence interval [CI]: 9.3–13.1) and 9.4 months (95% CI: 6.2–12.6), respectively, with no statistically significant difference (*P* = 0.176). There was no difference in the levels of mini-mental state examination score at 1, 3, and 6 months, as well as the risk of adverse events, after WBRT between the two groups. In conclusion, SEB may improve the remission rate of lesions but not prolong the OS time. The boost would neither increase serious side effects nor would it aggravate cognitive impairment caused by WBRT.

## 1 Introduction

Brain metastases (BMs) usually occur in the advanced stage of cancers and bring a significant cause of mortality among patients. The most common sources of BMs are lung, breast, and melanoma [1,2,3,4]. Most patients with BMs have a short life expectancy and a poor prognosis. The traditional treatments for BMs include surgery, radiotherapy, and chemotherapy, but their efficacy is facing bottlenecks in controlling the progression of intracranial lesions and prolonging life.

Immunotherapy recently has shown improved efficacy in comprehensively treating various solid tumors. Immunotherapy drugs, such as cytotoxic T-lymphocyte-associated protein 4 (CTLA-4) and immune checkpoint inhibitors (ICIs), have effectively prolonged the survival time of patients with advanced tumors [5,6,7,8], including patients with newly diagnosed BMs [2,5,9]. Brain radiotherapy, as a supplement to local treatment in the era of immunotherapy, has also been reported to control the progression of intracranial tumors and improve the related symptoms caused by BMs [9,10,11]. However, the current studies have numerous limitations. First, brain radiotherapy combined with immunotherapy may prolong progression-free survival (PFS) for patients with BMs, but with limited impacts on overall survival (OS). Second, most of the included patients had relatively good prognoses with no more than three BMs, and stereotactic radiosurgery (SRS) was utilized instead of whole brain radiotherapy (WBRT) to preserve cognitive function after radiotherapy. Thirdly, more than half of the patients used the CTLA-4 inhibitor.

In the real world, a considerable number of patients are diagnosed with multiple (three or more) BMs. WBRT, rather than SRS, should be the first choice, and the boost dose of radiation should be determined according to the specific location and size of BMs. Considering the influence of adverse drug responses and reimbursement policies, domestic patients are more likely to receive ICIs, rather than CTLA-4 inhibitors. The safety and efficacy of immunotherapy combined with WBRT in patients with multiple BMs, their effects on cognitive function, and the clinical difference of whether + sequential integrated boost (SEB) to BMs have not been reported. Herein, this retrospective study was conducted to explore appropriate modalities for local radiotherapy in multiple BMs in the immunotherapy era.

## 2 Methods

### 2.1 Study design and patients

This study retrospectively involved patients with BMs from 2021 to 2023, Department of Oncology (Radiotherapy), Kezhou People’s Hospital Affiliated to Nanjing Medical University. Inclusion criteria were as follows: (1) with definite pathological or histological diagnosis of primary tumor; (2) with diagnosis of multiple BMs (≥3) by brain magnetic resonance imaging (MRI) enhancement or positron emission tomography/computed tomography; (3) without primary intracranial tumor; and (4) the expected survival time ≥1 month. Exclusion criteria were as follows: (1) unable to cooperate with the cognitive function test due to stroke, mental disorders, or cognitive impairment caused by various reasons before treatment; (2) unable to tolerate brain radiotherapy and systemic immunotherapy due to severe underlying diseases; and (3) loss of follow-up.


**Informed consent:** Informed consent has been obtained from all individuals included in this study.
**Ethical approval:** The research related to human use has been complied with all the relevant national regulations and institutional policies and in accordance with the tenets of the Helsinki Declaration and has been approved by the Ethics Committee of the Affiliated Kezhou People’s Hospital of Nanjing Medical University (No. 2023-03-01).

### 2.2 Immunotherapy

All patients received immunotherapy with ICIs administered at least twice, and the drug was continued after the end of concurrent radiotherapy in conditional patients. The interval between the initiation of immunotherapy and the initial brain radiotherapy was no more than 1 week.

### 2.3 Radiotherapy

The patients were treated with a 6 MV X-ray from Elekta’s digital linear accelerator using lateral opposing fields for WBRT at a prescribed dose of 30 Gy delivered in 12 fractions over 3 weeks, with 5 fractions per week. According to whether to receive a boost, patients were allocated into the WBRT group (*n* = 27) to receive a dose of 30 Gy/12 fractions to primary lesions ≥1 cm and the WBRT + SEB group (*n* = 30) to receive a dose of 30 Gy/12 fractions +12–20 Gy/3–5 fractions to intracranial lesions showed by MRI. Important tissues and organs such as crystals, eyeballs, optic nerves, brain stem, and pituitary gland were in the safe dose range. Symptomatic treatments such as dehydration and intracranial pressure control were given in time during radiotherapy.

### 2.4 Curative effect and cognitive function

A contrast-enhanced MRI scan at 1 month after radiotherapy was performed to evaluate the changes of BMs according to the response evaluation criteria in solid tumors (RECIST 1.1). The lesions were classified as complete response (CR), partial response, stable disease, or progressive disease. The patients underwent follow-up every 3 months.

The cognitive function of patients was assessed using the mini-mental state examination (MMSE), which had a total score of 30 and was adjusted for education level. The first assessment MMSE scores within 7 days before treatment were used as the baseline level. The MMSE scores were measured at 1, 3, and 6 months after treatment (if the patient was alive and cooperative) and compared with the baseline level. The changes in cognitive function were divided into improvement, stability, or decline.

### 2.5 Statistical analysis

Statistical analyses were conducted using SPSS 22.0 software. The measurement data from the baseline patients were analyzed using a measurement analysis of variance and compared by two independent sample *t*-tests, the counting data were expressed in percentage or *n* (%) and were examined by a chi-square test. The effectiveness of multiple intracranial lesions and cognitive function was expressed as percentages or *n* (%) and was examined by chi-square test. The Kaplan–Meier survival analysis was used to compare the OS between the two groups.

## 3 Results

### 3.1 Characteristics of patients

Totally, 57 patients were enrolled, comprising 30 male and 27 female patients, whose mean age was 59 years. The prevalent diseases were primarily breast cancer, lung cancer, and malignant melanoma. No differences were found between the WBRT and WBRT + SEB groups in terms of age, gender, diagnosis of primary tumor, presence of intracranial lesions, KPS score, and type of immune medication at baseline (Table 1).

**Table 1:** Characteristics of study patients

Variables	WBRT group (*n* = 27)	WBRT + SEB group (*n* = 30)	*P*
**Gender,** ***n*** **(%)**			0.520
Male	13 (48.1)	17 (56.7)	
Female	14 (51.9)	13 (43.3)	
Age, years, Median (min, max)	60.0 (35–83)	57.5 (26–81)	0.468
**Primary disease,** ***n*** **(%)**			0.956
Non-small cell lung cancer*	19 (70.4)	19 (63.3)	
Malignant melanoma	3 (11.1)	3 (10.0)	
Breast cancer**	3 (11.1)	4 (13.3)	
Esophagus cancer	1 (3.7)	2 (6.7)	
Gastric cancer	1 (3.7)	2 (6.7)	
**Number of BMs,** ***n*** **(%)**			0.506
3–4	12 (44.4)	16 (53.3)	
≥5	15 (55.6)	14 (46.7)	
**Distant extracranial metastasis**			0.737
Yes	15 (55.6)	18 (60.0)	
No	12 (44.4)	12 (40.0)	
**KPS score,** ***n*** **(%)**			0.992
70	5 (18.5)	6 (20.0)	
80	16 (59.3)	16 (53.3)	
90	6 (22.2)	7 (23.3)	
100	0	1 (3.3)	
**Immune drugs,** ***n*** **(%)**			0.914
Pembrolizumab	2 (7.4)	1 (3.3)	
Camrelizumab	5 (18.5)	6 (20.0)	
Toripalimab	6 (22.2)	9 (30.0)	
Tislelizumab	6 (22.2)	8 (26.7)	
Sintilimab	7 (25.9)	5 (16.7)	
Durvalumab	1 (3.7)	1 (3.3)	

WBRT, whole brain radiotherapy; SEB, sequential integrated boost; BMs, brain metastases; KPS, Karnofsky performance status.

*In the WBRT group, 11 cases were squamous cell carcinoma, 8 cases were adenocarcinoma, and 5 cases had negative driver genes; in the WBRT + SEB group, 10 cases were squamous cell carcinoma, 9 cases were adenocarcinoma, and 5 cases had negative driver genes; driver genes mutations were progress after molecular targeted drug therapy, and/or had symptoms of brain metastasis.

**Triple-negative breast cancer in both groups.

### 3.2 Efficacy of intracranial lesions

**Figure 1:**
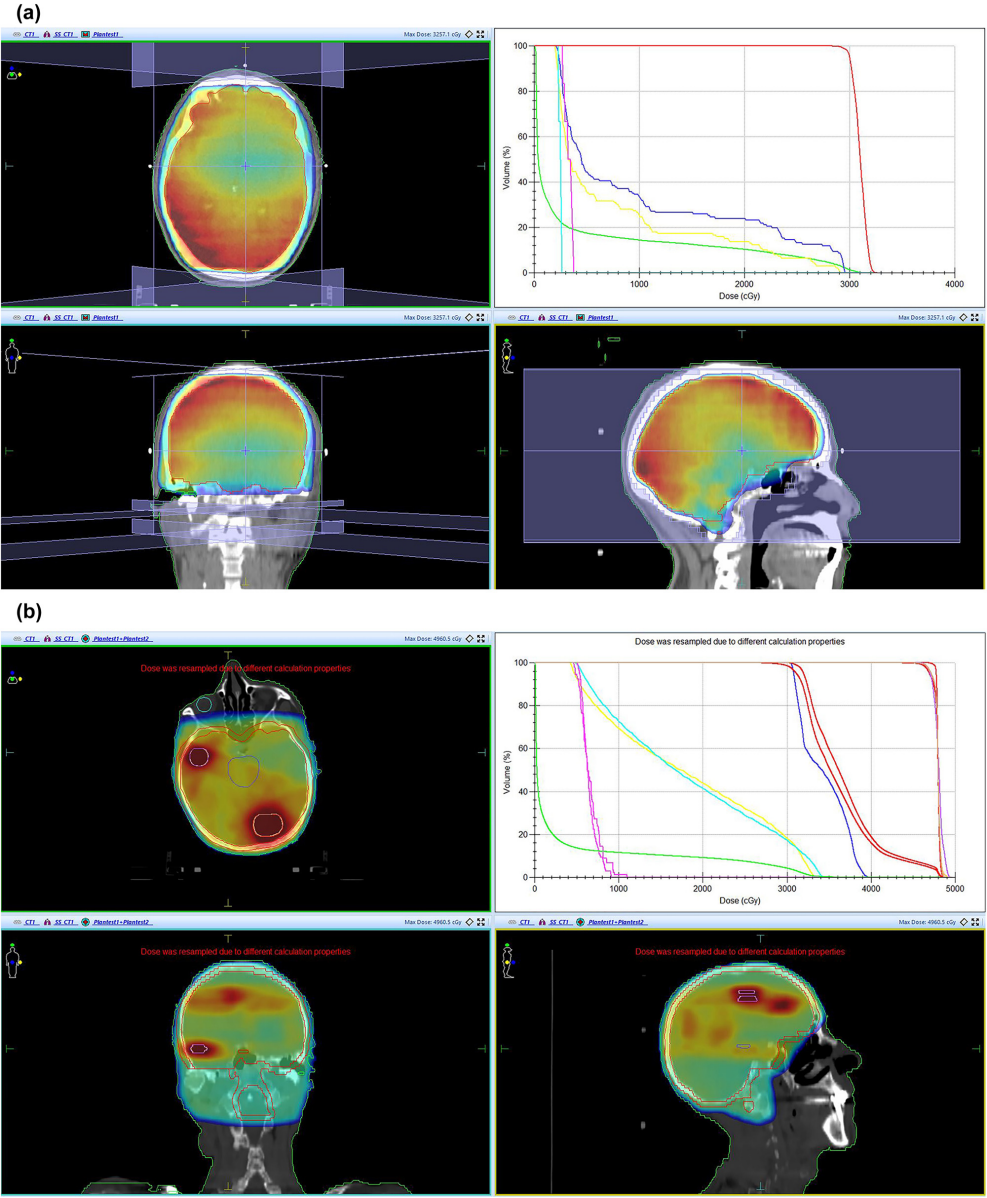
(a) WBRT scheme: A 63-year-old male patient with lung cancer and multiple BMS received lateral opposing fields at a prescription dose of 30 Gy/12 fractions (5 fractions per week). (b) WBRT + SEB scheme: A 66-year-old male patient with lung cancer received integrated boost at a dose of 4 Gy × 3 fractions to the lesions in temporal and occipital lobes after 30 Gy/12 fractions of WBRT, with a total dose of 46 Gy. Dose-volume histogram showed that the prescribed dose in the target area was up to the standard, and the important tissues and organs, including the brain stem, lens, and eyeball, were reasonably protected. WBRT, whole brain radiotherapy; SEB, sequential integrated boost.

The WBRT + SEB group had a significantly higher complete response rate (CRR) of intracranial lesions compared to the WBRT group (26.7 vs 14.8%, *P* < 0.05) at 1 month after radiotherapy. Compared with the WBRT group, SEB also improved the objective response rate (ORR) 1 month after radiotherapy (90.0 vs 66.7%, *P* = 0.032). Table 2 displays the specific efficacy of intracranial lesions, and Figure 1 shows the dose distribution of the groups and the doses to the surrounding important tissues.

**Table 2:** Efficacy of intracranial lesions

Group	CR	PR	SD	PD	*P*	ORR (%)	*P*
WBRT group (*n* = 27)	4	14	9	0	0.043	66.7	0.032
WBRT + SEB group (*n* = 30)	8	19	3	0		90.0	

CR, complete response; PR, partial response; SD, stable disease; PD, progressive disease; ORR, Objective Response Rate; WBRT, whole brain radiotherapy; SEB, sequential integrated boost.

### 3.3 OS

**Figure 2:**
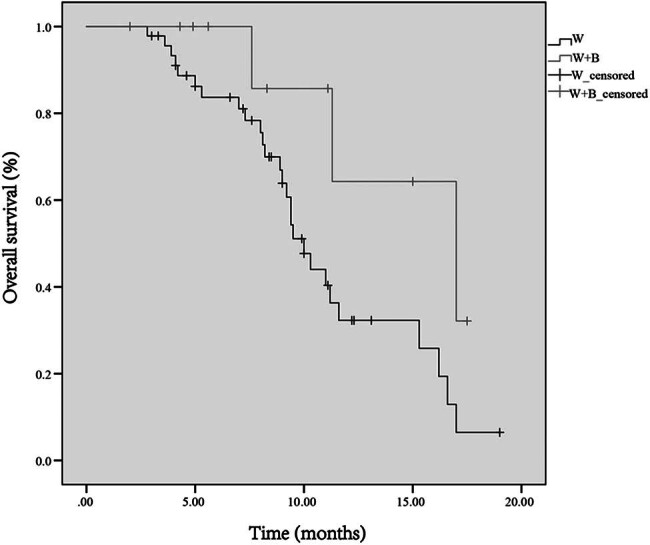
Survival curves of patients with multiple BMs treated with immunotherapy combined with WBRT and immunotherapy combined with WBRT + SEB BMs, brain metastases; WBRT, whole brain radiotherapy; SEB, sequential integrated boost.

During a median follow-up period of 16 months, the median OS for all 57 patients was 11.0 months. The median OS was estimated to be 9.4 months (95% confidence interval [CI]: 6.2–12.6) for the group receiving WBRT and 11.2 months (95% CI: 9.3–13.1) for the group receiving WBRT + SEB. No significant difference was found in OS between patients receiving WBRT and those receiving WBRT + boost (*P* = 0.176, hazard ratio [HR]: 0.607, 95% CI: 0.294–1.251) (Figure 2). The median OS time was estimated to be 17 months in patients with CR (95% CI: 8.3–25.7) and 10 months (95% CI: 8.9–11.1) in those without CR, with no statistical difference (*P* = 0.056).

### 3.4 Assessment of cognitive function

The MMSE was completed by all patients prior to the commencement of brain radiotherapy. At baseline, there was no difference in MMSE scores between the WBRT group and the WBRT + SEB group. The changes in cognitive function in both groups at 1, 3, and 6 months after radiotherapy are shown in Table 3, and the majority of patients achieve stability or improvement after treatment. A few patients showed delayed cognitive decline over time. No significant difference was found between the two groups in the proportion of survivors with cognitive decline at each time point (*P* > 0.05).

**Table 3:** Changes in cognitive function

Changes in MMSE scores, *n* (%)	End of radiotherapy		*P*	3 months after radiotherapy		*P*	6 months after radiotherapy		*P*
	WBRT group (*n* = 27)	WBRT + SEB group (*n* = 30)		WBRT group (*n* = 27)	WBRT + SEB group (*n* = 30)		WBRT group (*n* = 27)	WBRT + SEB group (*n* = 30)	
Improvement	5 (18.5)	4 (13.3)	0.793	3 (11.5)	3 (10.3)	0.780	2 (11.1)	3 (13.6)	0.714
Stability	14 (51.9)	15 (50.0)		11 (42.3)	15 (51.7)		8 (44.4)	12 (54.5)	
Decline	8 (29.6)	11 (36.7)		12 (46.2)	11 (37.9)		8 (44.4)	7 (31.8)	

MMSE, Mini-mental State Examination; WBRT, whole brain radiotherapy; SEB, sequential integrated boost.

### 3.5 Adverse events (AEs)

The main AEs were the symptoms caused by BMs and cranial irradiation, mainly including vertigo, headache, nausea, and fatigue, which were all mild to moderate, and were improved by dehydration and controlling intracranial pressure. The patients in the two groups had good tolerance and completed the treatment scheme, with no differences in all adverse reactions and no grade 4 AEs or above (Table 4).

**Table 4:** Major adverse reactions

Adverse reactions, *n* (%)	WBRT group (*n* = 27)	WBRT + SEB group (*n* = 30)	*P*
Headache	14 (51.9)	16 (53.3)	0.912
Nausea	11 (40.7)	13 (43.3)	0.843
Epileptic seizure	1 (3.7)	1 (3.3)	0.940
Vertigo	18 (66.7)	19 (63.3)	0.792
Paresthesia	4 (14.8)	5 (16.6)	0.849
Fatigue	15 (55.6)	16 (53.3)	0.866
Weight loss	4 (14.8)	4 (13.3)	0.873

WBRT, whole brain radiotherapy; SEB, sequential integrated boost.

## 4 Discussion

BMs, in contrast to extracranial metastases, are generally considered to be relatively less sensitive to immunotherapy [12], mainly because the brain tumor microenvironment contains numerous myeloid cells, while the number and proportion of CD8+ effector T lymphocytes are rather limited [13]. Under physiological conditions, it is difficult for peripheral immune cells to migrate into the brain parenchyma because the blood–brain and blood–cerebrospinal fluid barriers prevent the exchange of various macromolecules [14]. Although some studies suggested that immunotherapy can extend the life expectancy of patients with BMs to a certain extent [5,15], improving the sensitivity and effectiveness of immunotherapy in BM patients remains important [16].

Radiotherapy, an important means of treatment for BMs, can improve the effect of tumor immunotherapy by increasing tumor immunogenicity [17,18]. The synergistic mechanism of brain radiotherapy and immunotherapy includes that the changes in the permeability of the blood–brain barrier during radiotherapy promote peripheral immune cells to migrate into the brain tissue [19], and produce tumor-specific cytotoxic T lymphocytes [20,21,22]. Radiotherapy can affect the secretion of cytokines in the brain and enhance macrophages and dendritic cells in killing BMs, similar to the mechanism of radiotherapy on extracranial metastases. In addition to cytotoxicity, antigen presentation, phagocytosis [13], and triggering immune responses, radiotherapy can induce the abscopal effect, namely, localized radiotherapy can also lead to the regression of distant metastases after immunotherapy [23].

Kiess et al. treated BMs with brain radiotherapy combined with CTLA-4, with ideal intracranial lesion control (1-year regional recurrence-free survival rate: 64–92%), favorable OS outcome (1-year OS rate: 40–65%), and good tolerance [24]. However, not all studies have yielded consistent conclusions. Mathew et al. [25] and Patel et al. [26] found that despite the safety of brain radiotherapy combined with CTLA-4, the clinical benefit was modest, and no survival benefit was observed compared to patients with brain irradiation alone. In contrast, Xiao et al. [27] demonstrated that the combination of immunotherapy prolonged the PFS (HR = 0.48) and OS (HR = 0.64) of lung cancer patients with BMs compared with radiotherapy/chemotherapy alone. Differences in clinical findings may be interpreted by immunotherapy agents. A study found that the average OS of radiotherapy plus ICIs was significantly longer than that of radiotherapy plus CTLA-4 in patients with malignant melanoma and BMs, which was 27.4 and 7.5 months, respectively [28]. The research data of KEYNOTE-006 also acknowledged the higher security of ICIs than CTLA-4; therefore, ICIs were more widely used in metastatic melanoma [29]. In Xiao et al.’s study [27], ICIs combined with brain radiotherapy have also been confirmed to be effective and safe for lung cancer patients with BMs, most of the BMs regressed significantly, and the local and distant intracranial lesions were also controlled. Therefore, ICIs were used for all the immunotherapy in this study.

The remarkable thing is that most patients included in the studies mentioned above had few BMs and SRS. Because of relatively limited BMs and good tolerance to irradiation of a higher biological equivalent dose [30], these patients had a better prognosis than those with multiple BMs or diffuse BMs [9,31]. A previous small-sample retrospective study or a phase I study showed that WBRT + CTLA-4 for multiple BMs was safe but less effective than expected, with most patients experiencing progression of BMs and death, causing early termination of the studies [32,33]. In a study of patients with BMs from non-small cell lung cancer, the median PFS was significantly longer in patients undergoing WBRT combined with ICIs than those undergoing WBRT alone (11 vs 3 months, 95% CI: 6.3–15.6 vs 95% CI: 0.8–5.1), with 71% lower risk of disease progression (HR: 0.29, 95% CI: 0.11 0.80; *P* = 0.016) and an improved OS trend [10]. Similarly, most of the existing studies compared immunotherapy combined with brain radiotherapy with brain radiotherapy alone [4,34,35,36], while there were few reports comparing different radiotherapy methods under combination therapy. A meta-analysis revealed that the WBRT + ICI group had an ORR and disease control rate of 0 and 57%, respectively, for intracranial lesions, which were inferior to the SRS + ICI group’s 75 and 84%. The ORR and disease control rate of extracerebral tumors were also better in the SRS + ICI group (73 and 50%) than those in the WBRT combined with the ICI group (0 and 43%) [9]. However, it is important to point out that the meta-analysis included a limited number of studies, and patients who received WBRT had a higher tumor burden and worse prognosis, so there was a bias in the baseline.

Since there were few reports on the immunotherapy combined with WBRT for treating multiple BMs, this retrospective study was conducted and included patients who received WBRT with SEB. After reviewing these 57 cases, we found that patients with less than four residual lesions with a diameter greater than 1 cm after WBRT were more likely to be given a SEB. In addition, the different perspectives and choices of their oncologists were also important factors in determining whether a SEB after completing WBRT should be delivered. However, the baseline levels of the two groups were consistent. The median OS time was 11.2 and 9.4 months for the two groups, respectively, significantly better than the expected OS (5.5–10.0 months) of WBRT alone [31,37,38,39]. Although there was no significant improvement in OS in the WBRT + SEB group of this study, SEB could improve the CR and ORR of intracranial lesions, which may contribute to the longer patient survival in cases of multiple BMs. The survival data of the immunotherapy combined with WBRT + SEB group in this study were also consistent with reported data of brain radiotherapy (including SRS) combined with immunotherapy, such as a reduced risk of disease progression and death [4,34] and efficacy of controlling intracranial lesions [35].

In this study, immunotherapy and WBRT were administered concurrently, meaning there was no more than 1 week between the start of immunotherapy and the initial brain radiotherapy. Previous studies have shown that a time interval of more than 3 weeks between brain radiotherapy and immunotherapy can affect sensitivity. Relatively, an interval of no more than 2 weeks or less was not only safe and feasible but also improved BMs control, and 6-month and 1-year survival rates [35,40,41,42,43]. This phenomenon may be explained by changes in blood-brain barrier permeability as a result of brain radiotherapy, thereby enhancing the immune effect of ICI in the brain [44].

The doses of WBRT + SEB and fraction designing in this study were selected based on several previous studies [45,46,47,48,49,50]. A study involving 52 patients with BMs from lung cancer compared WBRT + SEB (WBRT: 30 Gy/10 fractions and BMs: 4 Gy × 3 fractions) with simultaneous WBRT + SEB (WBRT: 30 Gy/10 fractions and BMs: 40 Gy × 10 fractions). The study revealed that the survival rates at 1, 2, and 3 years were 60.0% compared to 47.8%, 41.1% compared to 19.1, and 27.4% compared to 0%, respectively, with the median survival time of 15 and 10 months, respectively; patients with less than 2 BMs had longer survival with SEB, and the MMSE score at 3 months after radiotherapy in the SEB group was higher than that in the SEB group [45]. The mechanisms driving improved SEB response rates were attributed to the higher equivalent biological dose effect and also resulted in better local control. Although WBRT + SEB significantly improved the response rate of BMs to radiation, not all the increasing radiotherapy dose in the above studies had translated SEB into a significant improvement in OS [48,49], which was consistent with the current study. It is important to note that the majority of patients in the aforementioned studies did not receive or were not reported to receive ICIs, and the numbers of BMs were not identical. SEB of 42–50 Gy may be safe for the right lesions (distance from the brain stem, crystal, optic nerve >2 cm). The total dose of WBRT was difficult to increase, which resulted in a higher risk of local recurrence. However, WBRT + SEB can effectively increase the dose of irradiation to target lesions, and the dose decay at the lesion edge would not increase the dose excessively to important tissue structures beyond 2 cm, thus safely and effectively making up for the deficiency of WBRT. The CRR of WBRT + SEB was significantly better than that of WBRT alone, which may further prolong the life expectancy of patients.

Brain radiotherapy, especially WBRT, on patients’ cognitive function is still a matter of debate. Reygagne et al. [51] believed that WBRT could effectively improve the prognosis of patients’ central nervous system. However, more papers reported that WBRT can cause damage to cognitive function, and the probability of a decline in cognitive function in patients with BMs in 3 and 12 months after WBRT was 31–57% and 48–89%, respectively. Therefore, SRS was more recommended for patients with less than 4 BMs [52]. In our study, the changes in cognitive function 1–6 months after brain radiotherapy were consistent [52]. Due to the large number of BMs in all patients, the radiotherapy methods adopted in this study were based on WBRT. In addition to some patients whose intracranial lesions were alleviated or effectively controlled, thus, cognitive function was improved; more than half of the patients had decreased or stable cognitive function during the 6-month follow-up after radiotherapy. Our study also showed that ICIs combined with WBRT + a limited boost dose did not aggravate cognitive impairment in patients with multiple BMs nor did it increase the risk of serious side effects.

There were some limitations in our study. First, this study had an insufficient sample size. Second, the PD-L1 expression levels were not detected in the retrospective study. Third, due to the large number and different distribution of BMs in the enrolled patients, the hippocampal structure was not specifically spared during radiation field design. Evaluation of the impact of radiotherapy and immunotherapy on treatment for BMs further needs to be separated into different tumor types to be meaningful. Validation through larger prospective studies with extended follow-up would be warranted.

## 5 Conclusion

In the present study, a combination of immunotherapy and WBRT was safe and feasible for patients with multiple BMs. On the basis of this combined therapy, SEB could improve the total resection rate of lesions but could not prolong the OS time. The boost may neither increase serious side effects nor aggravate cognitive impairment caused by WBRT.
